# ColonyTrak: a web tool and database system for managing experimental animal models

**DOI:** 10.1186/1471-2105-12-S7-A11

**Published:** 2011-08-05

**Authors:** Laasya Vadlamudi, Lynn A Jones, Ramin Homayouni

**Affiliations:** 1 Bioinformatics Program, University of Memphis, Memphis, TN, 38152, USA; 2 Department of Biology, University of Memphis, Memphis, TN, 38152, USA

## Background

Genetically modified animal models are useful for understanding mechanisms of human disease and for development of new therapeutics. Maintaining animal colonies and performing genetic experiments requires careful record keeping. Many commercial or publicly available data management tools exist for animal recordkeeping, however they are rather complicated and may require technical expertise to install and maintain [1,2]. Consequently, biologists often use spreadsheets or database tools that are tied to a single computer, which limits accessibility by multiple users at different locations. Here, we have developed a user-friendly tool called ColonyTrak, designed for the biologist, to enable data management from any remote computer using a web browser.

## Materials and methods

**Figure 1:**
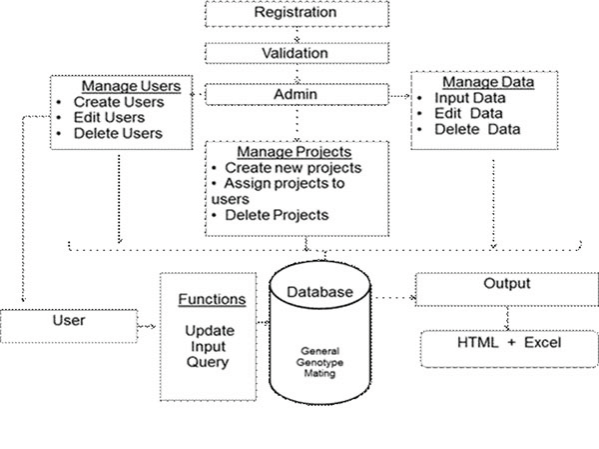
ColonyTrak workflow.

ColonyTrak was developed using a MySQL database, HTML (for GUI), CSS (for style sheets), PHP (server side scripting language), AJAX (for autofilling and auto increment features), and Javascript (for displaying alert messages or data validation) in a manner that is transparent to the end user. Registered users have administrator rights and, through a simple interface, are able to manage projects, users and read/write privileges (Figure 1). Users are able to enter a variety of animal data such as date of birth, genotype, lineage information, mating schemes from any location using a web browser. Several features such as auto-fill and auto-increment are implemented to make data entry easier.

## Results and conclusions

**Figure 2:**
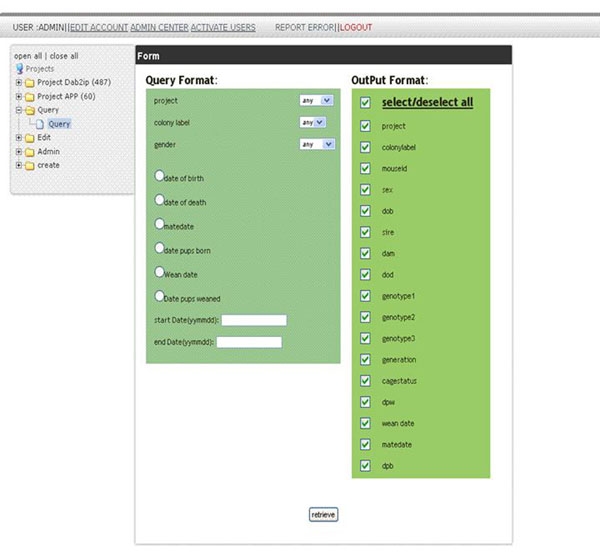
User specified query and output formats.

**Figure 3:**
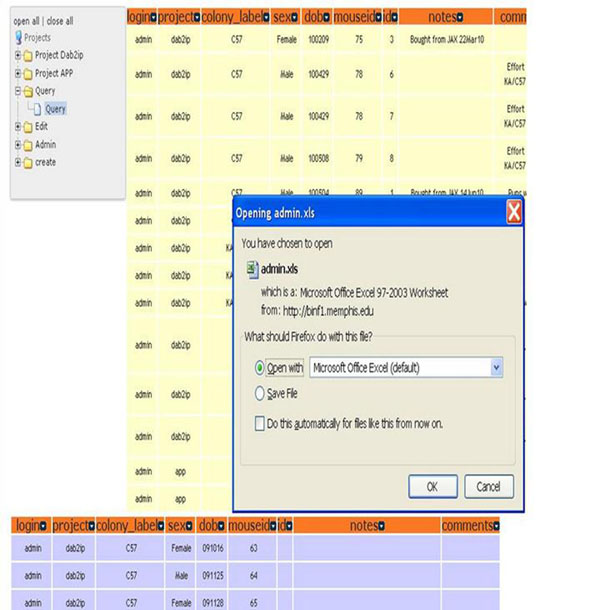
Tabular HTML and Excel output formats.

ColonyTrak provides a secure multi-level user environment to enter, store and retrieve animal data. Importantly, the tool allows multiple users to simultaneously query the database with simple or complex queries and export the results in tabular format. Queries can be formatted by using a combination of pull-down menus to select the colony label, project label and/or gender, followed by specification of the dates corresponding to date of birth, date of death, mating date, or date of weaning (Figure 2). In addition, the user can define specific output variables. The query results are displayed in tabular html format or can be downloaded in an Excel format (Figure 3). Animals which are in mating cages are displayed in the first table (tan color) followed by animals in holding cages (blue color). The values in each html column can be sorted by clicking on the column headings.

We posit that ColonyTrak will enhance research productivity and the efficiency with which biologist manage experimental animal models. ColonyTrak is available for free at http://binf1.memphis.edu/ColonyTrak/.
